# Reading High Breast Density Mammograms: Differences in Diagnostic Performance between Radiologists from Hong Kong SAR/Guangdong Province in China and Australia

**DOI:** 10.31557/APJCP.2020.21.9.2623

**Published:** 2020-09

**Authors:** Tong Li, Seyedamir Tavakoli Taba, Pek-Lan Khong, Tom X-L Tan, Phuong Dung (Yun) Trieu, Edward Chan, Moayyad E Suleiman, Ying Li, Patrick Brennan, Sarah Lewis

**Affiliations:** 1 *Breastscreen REader Assessment Strategy (BREAST), Medical Imaging Science, School of Health Sciences, Faculty of Medicine and Health, The University of Sydney, Sydney, Australia. *; 2 *Medical Imaging Science, School of Health Sciences, Faculty of Medicine and Health, The University of Sydney, Sydney, Australia. *; 3 *Department of Diagnostic Radiology, Faculty of Medicine, The University of Hong Kong, Hong Kong, China.*; 4 *Department of Medical Imaging, The University of Hong Kong-Shenzhen Hospital, Hong Kong, China. *

**Keywords:** Breast image reading, mammography, breast cancer screening, sensitivity, specificity

## Abstract

**Background:**

Variations in the performance of radiologists reading mammographic images are well reported, but key parameters explaining such variations in different countries are not fully explored. The main aim of this study is to investigate performances of Chinese (Hong Kong SAR and Guangdong Province) and Australian radiologists in interpreting dense breast mammographic images.

**Methods:**

A test set, contained 60 mammographic examinations with high breast density, was used to assess radiologists’ performance. Twelve Chinese and thirteen Australian radiologists read all the cases independently and were asked to identify all lesions and provide a grade from 1 to 5 to each lesion. Case sensitivity, specificity, lesion sensitivity, AUC and JAFROC were used to assess radiologists’ performances. Demographic information and reading experience were also collected from the readers. Performance scores were compared between the two populations and the relationships between performance scores and their reading experience were discovered.

**Results:**

For radiologists who were less than 40-year-old, lesion sensitivity, AUC and JAFROC were significantly lower in Chinese radiologists than those in Australian (52.10% vs 71.45%, p=0.043; 0.76 vs 0.84, p=0.031; 0.59 vs 0.72, p=0.045; respectively). Australian radiologists with less than 10 years of reading experience had higher AUC and JAFROC scores compared with their Chinese counterparts (0.83 vs 0.76, p=0.039; 0.70 vs 0.56, p=0.020, respectively).

**Conclusions:**

We found that younger Australian radiologists performed better at reading dense breast cases which is likely to be linked to intensive fellowship training, immersion in a screening program and exposure to the benefits of a performance-measuring education tool.

## Introduction

The incidence of female breast cancer has been increasing rapidly in China with an annual growth of 3-5% compared to Australia’s annual increase of 0.2% over the last 20 years (Li et al., 2016). In Hong Kong Special Administrative Region (SAR), an affluent area of People’s Republic of China (PRC), the age-standardised incidence rate of breast cancer has reached to 56.7/100,000 women in 2011-2015 (Wong et al., 2015). Given that decreased morbidity is highly reliant on the early detection of breast cancer, understanding diagnostic efficiency for breast cancer is paramount for positive health outcomes.

One of the most challenging features of reading screening mammograms is breast density and breasts in Asian women are, in general, very dense due to a high proportion of fibro-glandular tissue to fatty tissue (Bae and Kim, 2016). Dense breast tissue can mask the appearances of subtle cancers, microcalifications and architectural distortion (Suleiman et al., 2016a). The detection rate for breast cancer in screening mammograms with dense breast tissue is stubbornly low, and this difficulty is not currently recognised in education packages available internationally (Al Mousa et al., 2014). A recent study from the United States using volumetric breast density assessment predicted that mammography sensitivity dropped below 50% in its ability to detect cancers when the breast comprised of 25% volumetric breast density and above, which is even more important when considering Asian breast composition (Destounis et al., 2016).

Cancer detection also depends on individual readers’ interpretation of the mammographic images presented. In terms of error rates, perceptual errors in radiology can represent up to 63% of all diagnostic errors (Gandomkar et al., 2017). Reader characteristics are strongly linked to the minimisation of perceptual errors, with reading experience, reading volume and breast imaging specialisation being three key parameters (Rawashdeh et al., 2013). Feedback from Australian radiologists has suggested that test sets comprising of both practice and feedback tasks yield increased performance and confidence in early cancer detection (Suleiman et al., 2016b).

China has no population-based early detection screening program for breast cancer and a current barrier for the success of breast screening programs in China, including Hong Kong SAR, is two-fold: a lack of public confidence in the value of mammography screening for breast cancer and a lack of radiological expertise/training to build workforce capacity (Cancer Expert Working Group on Cancer Prevention and Screening, 2010). Traditionally, Chinese approaches to breast health and cancer have focused on the bio-medical model of treatment in late stage, with a paucity of research on the value of screening and cancer prevention. This has serious implications for public health as China is experiencing an ever-increasing incidence of breast cancer at twice as fast as global rates and is without a population-based screening program (Wang and Yu, 2015; Li et al., 2016). By comparison, Australia has had a national approach to breast cancer screening, known as BreastScreen Australia (BSA), since 1991 and there is clear evidence of a decrease in the mortality rate through this early detection system (Australian Institute of Health and Welfare, 2018). A recent scoping paper exploring the properties of Chinese and Australian breast imaging research found that the greatest divide in research strengths was the high focus on breast screening and breast density by Australian researchers compared with relatively low by Chinese researchers (Tavakoli Taba et al., 2019). 

The aim of this study is to understand the diagnostic efficiency of breast cancer in dense breasts using screening mammography by the reading performances between radiologists from Hong Kong SAR and Guangdong Province in China and Australia. This study represents an important comparison and learning opportunity between two regions that have close ties on a number of fronts. The number of Chinese migrants settling in Australia has doubled in the last decade and are now the largest national group seeking permanent migration. China is also Australia’s largest trading partner and a key focus of economic and collaboration in what is being called the “Asian century” (Commonwealth of Australia, 2012). As the demographics of Australia’s aging population change to reflect its higher Asian migration policies over the last three decades, it is timely to investigate the performance of two different yet converging breast imaging services that aim to support cancer diagnosis in their respective populations. 

## Materials and Methods

*Study design*

This study used a mammographic test set from Breastscreen REader Assessment STrategy (BREAST) program to assess radiologists’ performance in Hong Kong SAR/Guangdong Province in China and Australia. The BREAST program was developed by The University of Sydney and Cancer Institute New South Wales, and financially supported by Cancer Institute New South Wales and Department of Health and Aging in Australia, to assess readers’ performance and ultimately enhance diagnostic efficiency of breast cancer through educational packages (Brennan et al., 2013). Ethical approval was granted for this study (Human Research Ethics Committee of the University of Sydney: 2017/028) and informed consent was obtained from each reader at test set reading. 

*Participants*

A total of 25 radiologists (12 in Hong Kong SAR/Guangdong Province in China and 13 in Australia) participated in this study. All the participants are certified radiologists in their respective countries and an identical test set was read by radiologists from both regions. Each reader’s information was collected through a web-based questionnaire, including year of birth, year of reading experience, number of examinations read per week and number of hours reading per week. All data were de-identified. 

Australian participants include readers who registered for the BREAST workshop at the Royal Australian and New Zealand College of Radiology (RANZCR) Annual Scientific Meeting in 2018. Chinese participants included readers who were actively employed in breast imaging in hospitals and private clinics in Hong Kong SAR and at a large referral hospital in Shenzhen, Guangdong Province, PRC. These Chinese radiologists were recruited via voluntary methods onsite at their workplace.

*Image test set*

The test set contained 60 mammographic examinations extracted from the digital archives of BSA under a research agreement. Of those 60 cases, 20 contained a biopsy-proven cancer case consisting of a mixture of stellate masses, spiculated masses, discrete masses, calcifications, architectural distortion and non-specific density. A total of 21 lesions could be located in the 20 cancer cases, including 19 cases contain only one lesion for each case and the other one case contains 2 lesions (1 in left and 1 in right side). The remaining 40 examinations were normal cases verified by two experienced breast radiologists working for BSA with a 2-year negative screening follow-up. Each examination consisted of a standard two-view mammograms (cranio-caudal and medio-lateral oblique projections) of both breasts obtained from full field digital mammography as well as a two-view images of a previous screening round where available (35/60 cases). 

All of the 60 cases were selected from screening women who had dense breasts, simulating an environment that may be found in an Asian breast screening environment. Dense breasts were defined as being reported as either category 3 (51-75% glandular) or category 4 (> 75% glandular) according to RANZCR synoptic breast imaging report (National Breast Cancer Centre, 2007). 

*Reading environment*

The reading environments were standardised for all of the radiologists. All mammograms were in DICOM format and were displayed on two 5-megapixel monitors using local Picture Archiving and Communication System. The online-based assessment software, comprising of the identical images but in JEPG format, presented breast images on a full native resolution. Participants from Hong Kong SAR and Guangdong Province in China completed their test reading in the place of their employment under supervision of the research team in a vacant reading room in July 2018. One of the research team was a native Mandarin speaker in order to assist with any small translation requirements as instructions were given in English. Australian participants undertook the test set in a reading room that simulated a clinical radiology environment at the RANZCR Annual Scientific Meeting in Canberra, October 2018.

*Performance assessment*

Each radiologist read the test set independently without any awareness of the prevalence of cancer in the test set. For each case, participants were asked to identify and localise all detected lesions as well as provide a grade to each lesion in accordance with RANZCR Imaging Classification (1 - No significant abnormality; 2 – Benign; 3 – Indeterminate/equivocal; 4 – Suspicious; 5 - Malignant). For lesions with rating greater than 2, radiologists were also asked to classify the lesion type using a drop-down menu. The maximum reading time given to each participant was 2.5 hours. 

At the completion of reading, performance metrics including sensitivity, specificity, lesion sensitivity, Area under the Receiver Operating Characteristic curve (AUC) and Jackknife Free-response Receiver Operating Characteristic (JAFROC) Figure-of-Merit, were calculated and presented to each radiologist. Readers could scroll through their decisions and receive feedback if they wished to do so. The definition or description of each metric was shown to each radiologist at the conclusion of their reading session to allow them to understand their BREAST feedback (BreastScreen Reader Assessment Strategy, 2019):

- Sensitivity: the proportion of positive cases that were correctly called positive (i.e. rating a case 3, 4, or 5);

- Specificity: the proportion of negative cases that were correctly called negative (i.e. rating a case 1 or 2);

- Lesion Sensitivity: the proportion of individual lesions that were correctly identified and located;

- Area under the receiver operating characteristic curve (AUC): acquired by combining case sensitivity, specificity and confidence ratings;

- Jackknife free-response receiver operating characteristic (JAFROC) figure-of-merit: acquired by combining lesion sensitivity, specificity and ratings. 

Calculations of AUC and JAFROC were based on Dr. Chakraborty’s publications and formulae (Chakraborty, 2005; Chakraborty, 2017).

*Statistical analysis*

Age and years of reading experience were collected as continuous variables, but we used the cut-off points from receiver operating characteristic analysis to recode these two variables into categorical variables with two groups (above or below the cut-off point). Number of reading hours per week (<20, 20-60, 61-100, 101-150, 151-200, >200 hours/week) and number of cases read per week (<5, 5-10, 11-15, 16-20, 21-30, >30 cases/week) were recoded into dichotomous variables due to limited number in each category. Overall performance values and other characterises were compared between two regions using Mann-Whitney U test. SPSS (IBM SPSS statistics for windows, version 24.0) statistical package was used for all statistical analyses, and two-tailed tests of significance were employed using a significance level of 0.05.

## Results

*Overall performance*

**Figure 1:**
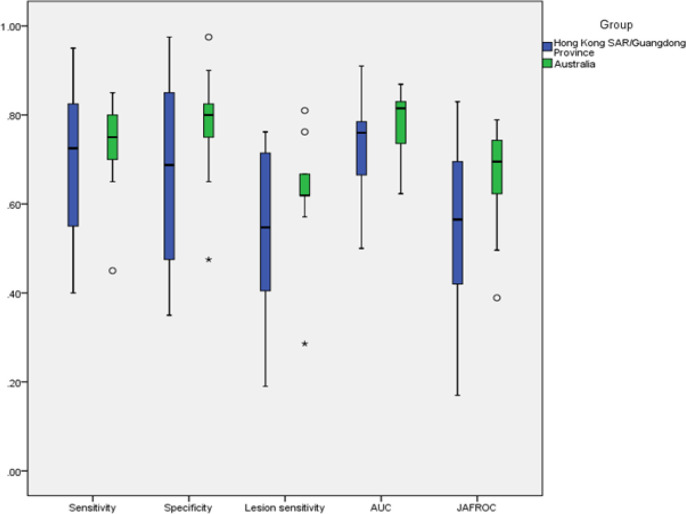
Boxplots of Radiologists’ Performances in Hong Kong SAR/Guangdong Province and Australia

The range of every performance metric was large for Hong Kong SAR/Guangdong Province radiologists compared to Australian ones as it is presented in Figure 1. However, the overall performance for radiologists in Hong Kong SAR/Guangdong Province and Australia did not show any difference in terms of sensitivity (p = 0.805), specificity (p = 0.340), lesion sensitivity (p = 0.216), AUC (p = 0.174) and JAFROC (p = 0.092). The descriptive data were presented in supplementary Table 1.

*Performance by age*

The median age of radiologists in Hong Kong SAR/Guangdong Province was significantly lower than that in Australia (37 vs 46, p = 0.038). For radiologists who were less than 40 years old, lesion sensitivity, AUC and JAFROC were significantly lower in Hong Kong SAR/Guangdong Province than those in Australia (Table 1). 

*Performance by reading experience*

No difference was found between the medians of years of mammographic reading experience in the two regions (6 vs 14, p = 0.090). However, Table 2 showed that for those who had reading experience of less than 10 years, Australian radiologists had higher AUC and JAFROC scores compared with their counterparts in Hong Kong SAR/Guangdong Province. 

There was no difference between radiologists’ performances for various workloads, except that the lesion sensitivity was lower in Hong Kong SAR/Guangdong Province for radiologists who read 10 hours per week and less (Table 3 and Table 4). 

**Table 1.** Comparison of Medians (Range) of Radiologists’ Performance in Different Age Groups in Hong Kong SAR/Guangdong Province and Australia

	Sensitivity (%)	Specificity (%)	Lesion sensitivity (%)	AUC	JAFROC
Who were < 40-year-old					
Hong Kong SAR/Guangdong Province (9 readers)	65.00 (55.00)	60.00 (62.50)	52.10 (57.20)	0.76 (0.39)	0.59 (0.54)
Australia (4 readers)	82.50 (5.00)	78.75 (5.00)	71.45 (14.30)	0.84 (0.05)	0.72 (0.13)
*P*-value	0.209	0.393	0.043	0.031	0.045
Who are ≥ 40-year-old					
Hong Kong SAR/Guangdong Province (3 readers)	80.00 (35.00)	87.50 (47.50)	66.70 (38.10)	0.76 (0.22)	0.54 (0.30)
Australia (9 readers)	70.00 (35.00)	80.00 (50.00)	61.90 (38.10)	0.77 (0.22)	0.66 (0.36)
*P*-values	0.349	0.853	0.393	0.926	0.926

Range, the difference between the highest and the lowest values; AUC, Area under the Receiver Operating Characteristic curve; JAFROC, Jackknife Free-response Receiver Operating Characteristic.

**Table 2:** Comparison of Medians (Range) of Radiologists’ Performance with Different Reading Experience in Hong Kong SAR/Guangdong Province and Australia

	Sensitivity (%)	Specificity (%)	Lesion sensitivity (%)	AUC	JAFROC
Reading experience < 10 years					
Hong Kong SAR/Guangdong Province (8 readers)	72.50 (55.00)	57.50 (57.50)	54.75 (57.20)	0.76 (0.39)	0.56 (0.54)
Australian (6 readers)	80.00 (20.00)	80.00 (15.00)	66.70 (23.90)	0.83 (0.10)	0.70 (0.13)
*P*- values	0.393	0.173	0.134	0.039	0.02
Reading experience ≥ 10 years					
Hong Kong SAR/Guangdong Province (4 readers)	72.50 (45.00)	82.50 (55.00)	57.15 (38.10)	0.74 (0.22)	0.62 (0.41)
Australian (7 readers)	70.00 (35.00)	75.00 (50.00)	61.90 (38.10)	0.74 (0.21)	0.62 (0.36)
*P*- values	0.703	0.705	0.771	0.85	0.85

Range, the difference between the highest and the lowest values; AUC, Area under the Receiver Operating Characteristic curve; JAFROC, Jackknife Free-response Receiver Operating Characteristic.

**Table 3:** Comparison of Medians (Range) of Performance of Hong Kong SAR/Guangdong Province and Australian Radiologists with Different Reading Hours Per Week

	Sensitivity (%)	Specificity (%)	Lesion sensitivity (%)	AUC	JAFROC
Who read ≤ 10 hours per week					
Hong Kong SAR/Guangdong Province (7 readers)	60.00 (55.00)	80.0 (57.50)	12.90 (57.20)	0.69 (0.39)	0.54 (0.53)
Australia (10 readers)	72.50 (40.00)	76.25 (50.00)	61.90 (52.40)	0.77 (0.25)	0.66 (0.40)
*P*-values	0.238	0.769	0.049	0.283	0.051
Who read > 10 hours per week					
Hong Kong SAR/Guangdong Province (5 readers)	80.00 (30.00)	60.00 (55.00)	66.70 (23.80)	0.76 (0.19)	0.69 (0.41)
Australia (3 readers)	80.00 (15.00)	80.00 (0.00)	66.70(9.50)	0.83 (0.04)	0.75 (0.06)
*P*-values	0.759	0.169	0.638	0.169	0.297

Range, the difference between the highest and the lowest values; AUC, Area under the Receiver Operating Characteristic curve; JAFROC, Jackknife Free-response Receiver Operating Characteristic

**Table 4:** Comparison of Medians (Range) of Performance of Hong Kong SAR/Guangdong Province and Australian Radiologists with Different Number of Reads Per Week

	Sensitivity (%)	Specificity (%)	Lesion sensitivity (%)	AUC	JAFROC
Who read < 60 cases per week					
Hong Kong SAR/Guangdong Province (6 readers)	62.50 (55.00)	81.25 (57.50)	47.65 (42.90)	0.74 (0.28)	0.57 (0.34)
Australia (7 readers)	75.00 (40.00)	80.00 (50.00)	61.90 (38.10)	0.78 (0.20)	0.66 (0.35)
*P*-values	0.429	0.943	0.221	0.568	0.199
Who read ≥ 60 cases per week					
Hong Kong SAR/Guangdong Province (6 readers)	80.00 (45.00)	57.50 (55.00)	64.30 (57.20)	0.76 (0.41)	0.61 (0.66)
Australia (6 readers)	72.50 (20.00)	77.50 (25.00)	66.70 (23.90)	0.84 (0.15)	0.72 (0.23)
*P*-values	0.627	0.127	0.464	0.333	0.262

Range, the difference between the highest and the lowest values; AUC, Area under the Receiver Operating Characteristic curve; JAFROC, Jackknife Free-response Receiver Operating Characteristic.

## Discussion

This study showed diagnostic efficacy for breast cancer was similar in Hong Kong SAR/Guangdong Province in China and Australia in terms of overall performance when both groups of radiologists read an identical mammographic test set. Even though there was no statistical significance in the medians of the five performance metrics between two regions, the ranges and interquartile ranges of these metrics were much more widely distributed for radiologists in Hong Kong SAR/Guangdong Province. This was a surprising finding as we had theorised that Chinese radiologists would be more accustomed to viewing high density breast cases in short, intense reading times. The lack of statistical difference was likely due to the high diagnostic performance of a small number of experienced radiologists from Hong Kong SAR/Guangdong Province, and when the results were analysed according to age and years of experience, difference in performance was evident between Australian and combined Hong Kong SAR/Guangdong Province readers. When considering the higher performance of AUC and JAFROC for all Australian readers (although not statistically significant), and especially for those Australian participants younger than 40 years of age, it is reasonable to report that Australian radiologists were better at reading high density mammograms, and particularly developed these skills at a younger age.

Radiologists’ age is not generally considered to be related to reading performance (Rawashdeh et al., 2013). However in our study, we found that younger Australian radiologists performed better at reading dense breast cases with 39% and 40% higher at lesion sensitivity and JAFROC values compared to their counterparts in Hong Kong SAR/Guangdong Province. This could be partially caused by a larger proportion of younger Australian radiologists having undertaken a breast imaging fellowship (3-6 months) than radiologists in Hong Kong SAR/Guangdong Province. We found an extremely strong association between lesion sensitivity and fellowship training in younger radiologists in our study, indicating that the dedicated system of specialised breast education, likely through BSA but also through exposure to educational platforms such as BREAST, were working favourably. Our finding is consistent with previous studies regarding intensive education (Elmore et al., 2009) and although the fellowship-match-performance was not significant (p = 0.058) in younger Australian readers, we believe this result may become more important with greater participant numbers. Even though many studies highlighted that radiologists’ performance improved with reading experience (Miglioretti et al., 2007; Reed et al., 2010), our study did not show any relationship between years of experience of mammograms reading and lesion sensitivity, AUC and JAFROC scores (all p > 0.05) in radiologists younger than 40-year-old. This finding might estimate that, compared to reading experience, fellowship training may be of greater benefit to younger readers and subsequently enhance their performance. This finding has important implications for expert capacity building in countries like PRC where early detection programs are not yet established.

One other possible explanation for the performance differences in younger readers is the lack of population-based breast screening in China, including Hong Kong SAR. In particular, lesion sensitivity refers to the ability to precisely locate lesions in the breast, and it is suggested to be associated with size of the lesion, with larger lesions being more visible (Mello-Thoms et al., 2014). A national, multi-centre study in China found that breast cancer tumours diagnosed in a non-screening/diagnostic environment were found to be in large size (> 30mm) in Chinese women, and this would be typically classified as late stage diagnosis in Australia (Li et al., 2011). The lesion size detected in breast screening programs is expected to be smaller than that diagnosed in diagnostic settings as the focus is on early diagnosis and asymptomatic women. As revealed in our study, younger radiologists in Hong Kong SAR/Guangdong Province had more difficulty reporting the location of cancer lesions accurately, and this may be attributed to the masking effect of breast density although these readers would be constantly exposed to high density cases. In contrast, with the majority of the Australian radiologists in this study being BSA readers, it is suggested that they are more skilled in detecting small tumours in asymptomatic women. This is likely to have contributed to the higher value of lesion sensitivity and the subsequent effects on JAFROC metric. 

Another interesting finding in our work is the differences in AUC and JAFROC values between two groups of radiologists with reading experience of less than 10 years. Again, better performance was found from Australian radiologists, and this may be attributed to the feedback mechanisms that support social learning within both structured screening programs (Taba et al., 2017) and through dedicated educational initiatives (Poot and Chetlen, 2016). To our knowledge there is no such assessment and training modules in Hong Kong SAR/Guangdong Province and there is no population-based screening program for breast cancer. The development of interactive training and education programs is likely to improve diagnostic efficiency, particularly for radiologists with lower levels of experience and less dedicated time devoted to image reading. 

There are a few limitations in our study. It is noted that the sample size in both groups are relatively small and further study with more participants to strengthen findings is required, especially for results which were on the cusp of significance. We were also unable to examine if there was any interactions between younger age and less experience for two regions due to the insufficient data in sub-groups. In this paper we have grouped radiologists from Hong Kong SAR and radiologists from Guangdong Province together and acknowledge that there are difference in education and work responsibilities that are not the same although some staff have shared managerial responsibility. It is likely with greater numbers to have a clearer picture of readers’ performance from Hong Kong SAR versus mainland China although there was no statistical significant difference in the performance metrics between these two regions in this study (all p > 0.05). Furthermore, it is important to acknowledge that this work is based on a test set methodology to examine the readers’ performance for cancer detection, which may not fully represent the diagnostic efficiency in both regions compared to studies using clinical auditing data as details about the clients were not given. 

In conclusion, our work showed that the overall diagnostic efficiency in reading test sets of breasts by Hong Kong SAR/Guangdong Province radiologists was similar to that of an Australia reader sample. However better performance was evident for locating breast lesions by younger Australian radiologists compared to their Hong Kong SAR/Guangdong Province counterparts. This disparity is likely to be linked to intensive fellowship training, immersion in a screening program and exposure to the benefits of a performance-measuring education tool such as BREAST. A lack of a similar supportive system, especially a lack of breast screening program in Hong Kong SAR/Guangdong Province has resulted in lower performance in localising lesions in dense breast tissue despite strong familiarity in reading these more difficult images. We believe our preliminary findings provide an initial understanding of breast diagnosis in Hong Kong SAR/Guangdong Province and their differences from Australia and has identified strategies to improve cancer care at an early detection stage for both women with breast cancer but also for the wider screened population.
